# Imaging for cardiac resynchronisation therapy requires cardiac magnetic resonance

**DOI:** 10.1007/s12471-018-1195-0

**Published:** 2018-11-07

**Authors:** P. Bhagirath, A. Zweerink, C. Allaart, M. Götte

**Affiliations:** Department of Cardiology, Amsterdam University Medical Center—VUmc, Amsterdam, The Netherlands

Recently, Ngyuen et al. tested the technical feasibility of using computed tomography angiography (CTA) for visualisation of the coronary venous anatomy in a limited number of patients scheduled for cardiac resynchronisation therapy [1]. In 15 patients a cardiac resynchronisation therapy device was implanted. Based on their findings, the authors conclude that a pre-procedural CTA may be beneficial for cardiac resynchronisation therapy implantation by identifying additional veins.

It is well recognised that a successful response to cardiac resynchronisation therapy depends on appropriate patient selection. For this purpose, comprehensive evaluation, including functional assessment, determination of electrical or mechanical synchrony and/or dyssynchrony, presence and location of fibrotic tissue, and visualisation of the coronary venous anatomy are crucial.

As indicated by the authors, cardiac magnetic resonance imaging (CMR) is often applied in patients considered for cardiac resynchronisation therapy. The authors themselves used CMR for the assessment of global left ventricular function. However, CMR is not only the gold standard for the evaluation of global function, but also provides other distinctive characteristics relevant to cardiac resynchronisation therapy, such as quantification of regional mechanical function by means of feature tracking or tagging analysis and identification of “healthy” (non-fibrotic) tissue in the lateral left ventricular myocardium which may serve as a target area for left ventricular lead implantation.

In addition, it has been demonstrated almost a decade ago that CMR can accurately visualise coronary venous anatomy [2]. Furthermore, Shetty et al. demonstrated the feasibility of merging CMR-derived vein anatomy with dyssynchrony maps and tissue characteristics peri-procedurally for guidance of cardiac resynchronisation therapy implantation [3]. This integrated, CMR-assisted cardiac resynchronisation therapy implantation resulted in improved clinical outcome, with a significantly higher long-term response to cardiac resynchronisation therapy.

More recently, CE-marked CMR-compatible cardiac resynchronisation therapy devices have been launched for clinical applications. These novel devices not only offer a standardised, single-modality work-up of candidates for cardiac resynchronisation therapy, but also allow for a more uniform and comprehensive follow-up. These improvements will stimulate new research including the evaluation of pacing effects on the myocardium using CMR, which may contribute to individualised optimisation of cardiac resynchronisation therapy.

Therefore, based on its unique capabilities, CMR can be considered the preferred imaging modality for patient-tailored cardiac resynchronisation therapy. To what extent solitary visualisation of coronary sinus anatomy based on CTA will add clinical value remains unclear.
